# Supplementary material for: Amendments to saline-sodic soils showed long-term effects on improving growth and yield of rice (Oryza sativa L.)
Source: PeerJ. 2020 Mar 10;8:e8726. doi: 10.7717/peerj.8726 (PMC7069413; doi:10.7717/peerj.8726)
Supplement: Supplemental Information 5 — SW-Na+ content, Na+ content in soil solution (1:5 soil to water extracts); SW-K+ content, K+ content in soil solution (1:5 soil to water extracts); SW-Ca2+ content, Ca2+ content in soil solution (1:5 soil to water extracts); S-Na+ content, Na+ content in plant shoot; S-K+ content, K+ content in plant shoot; S-Na+ content, Na+ content in plant root; S-K+ content, K+ content in plant root; W-Na+ content, Na+ content in the whole plant; W-K+ content, K+ content in the whole plant; SA, selective absorption of K+ over Na+; ST, selective transport of K+ over Na+ [file peerj-08-8726-s005.docx]

Table S1 Results of ANOVAs for the effects of treatment and block on rice plant and soil characteristics.

|  | Factors | *d.f.* | SW-Na^+^ content | | SW-K^+^ content | | SW-Ca^2+^ content | |  |  |
| --- | --- | --- | --- | --- | --- | --- | --- | --- | --- | --- |
| Soil |  |  | F | *P* | F | *P* | F | *P* |  |  |
|  | Treatment | 4 | 4.045 | 0.044 | 0.747 | 0.587 | 1.726 | 0.237 |  |  |
|  | Block | 2 | 0.188 | 0.832 | 5.689 | 0.029 | 0.079 | 0.925 |  |  |
| Rice yield |  |  | Rice yield (2010) | | Rice yield (2012) | | Rice yield (2015) | | Rice yield (2017) | |
|  |  |  | F | *P* | F | *P* | F | *P* | F | *P* |
|  | Treatment | 4 | 12.297 | 0.002 | 8.339 | 0.006 | 9.292 | 0.004 | 1.663 | 0.25 |
|  | Block | 2 | 0.343 | 0.72 | 0.204 | 0.82 | 2.023 | 0.195 | 1.132 | 0.369 |
| Rice plant |  |  | S-Na^+^ content | | S-K^+^ content | | R-Na^+^ content | | R-K^+^ content | |
|  |  |  | F | *P* | F | *P* | F | *P* | F | *P* |
|  | Treatment | 4 | 2.162 | 0.164 | 6.626 | 0.012 | 0.39 | 0.81 | 3.092 | 0.082 |
|  | Block | 2 | 2.803 | 0.119 | 1.281 | 0.329 | 2.367 | 0.156 | 0.8 | 0.482 |
|  |  |  | W-Na^+^ content | | W-K^+^ content | | SA | | ST | |
|  |  |  | F | *P* | F | *P* | F | *P* | F | *P* |
|  | Treatment | 4 | 0.907 | 0.503 | 6.826 | 0.011 | 2.613 | 0.115 | 1.758 | 0.23 |
|  | Block | 2 | 1.174 | 0.357 | 2.432 | 0.15 | 3.619 | 0.076 | 3.282 | 0.091 |

Note: SW-Na^+^ content, Na^+^ content in soil solution (1: 5 soil to water extracts); SW-K^+^ content, K^+^ content in soil solution (1: 5 soil to water extracts); SW-Ca^2+^ content, Ca^2+^ content in soil solution (1: 5 soil to water extracts); S-Na^+^ content, Na^+^ content in plant shoot; S-K^+^ content, K^+^ content in plant shoot; S-Na^+^ content, Na^+^ content in plant root; S-K^+^ content, K^+^ content in plant root; W-Na^+^ content, Na^+^ content in the whole plant; W-K^+^ content, K^+^ content in the whole plant; SA, selective absorption of K^+^ over Na^+^; ST, selective transport of K^+^ over Na^+^.
